# Facile Preparation of Crosslinked PAN Membranes Based on Thiol-Ene Photopolymerization

**DOI:** 10.3390/polym9090390

**Published:** 2017-08-28

**Authors:** Zhengdong Fei, Tao Wang, Ping Fan, Feng Chen, Mingqiang Zhong

**Affiliations:** College of Materials Science and Engineering, Zhejiang University of Technology, Hangzhou 310014, China; feizd@zjut.edu.cn (Z.F.); wangtao@zjut.edu.cn (T.W.); fanping@zjut.edu.cn (P.F.); Chenf@zjut.edu.cn (F.C.)

**Keywords:** polyacrylonitrile, membranes, thiol-ene click reaction, crosslinking, UV irradiation

## Abstract

To improve the mechanical strength and antipollution properties of membranes, this research presents a facile method to prepare crosslinked polyacrylonitrile (PAN) membranes. This was achieved firstly by radical copolymerization with acrylonitrile, allyl methacrylate and sulfobetaine methacrylamide. Then, the copolymer was crosslinked by a thiol-ene click reaction under UV irradiation. Finally, the crosslinked membranes were prepared by traditional immersion precipitation phase inversion. These prepared membranes showed excellent water-pressure resistance and solvent swelling, owing to their crosslinked structure. This research will help in preparing crosslinked membranes through facile crosslinking under mild reaction conditions. The betaine structure also considerably improved the antifouling properties of the membranes.

## 1. Introduction

Membrane technology develops rapidly, and has been widely applied in many fields such as water treatment, chemical reagent distillation, gas separation and protein purification [1,2,3]. For all these processes, the development of sophisticated membrane technology with controllable separation performance, long-term stability and the conservation of desired functions is very critical and promising [4,5]. However, it is difficult for the unmodified pristine membranes to simultaneously meet the requirements of mechanical strength, separation efficiency, cost and some functionality such as responsive membrane separation [6]. Therefore, it is necessary to develop facile modification strategies and design more reasonable membrane structures to obtain optimal properties [7,8,9].

Membrane processes such as ultrafiltration (UF), nanofiltration (NF) and reverse osmosis (RO) are the most effective strategies for freshwater augmentation. However, traditional membrane processes operate under high pressure, and therefore, the membranes used should have good mechanical strength. At the same time, the thickness of membranes can be decreased for better water flux if the membranes’ mechanical strength is high enough. On the other hand, for stable structures, low swelling is very important to treat wastewater with high concentrations of organic compounds. Two measures have been taken to enhance the mechanical strength of membranes: using high strength materials such as polysulfides, aromatic polymers, or polyamides, and preparing composite membranes. However, both methods increase the water-treatment cost [10,11].

Here, we present a facile crosslinking technique under UV irradiation by using the thiol-ene click reaction to prepare a crosslinked PAN membrane. This crosslinking process can be completed within 1 min under UV irradiation [12,13,14]. Therefore, the crosslinking process can be combined with immersion precipitation phase inversion (IPPI). The mechanical strength, water-pressure resistance and resistance to organic solvents of the crosslinked membranes were investigated. Betaine, a hydrophilic zwitterion, was also introduced into the membranes to improve their antipollution ability. To our best knowledge, this is the first report on the preparation of crosslinked membranes by an in situ crosslinking reaction.

## 2. Experimental

### 2.1. Materials

Acrylonitrile (AN, 98%), allyl methacrylate (AMA, 98%), 1-cysteine hydrochloride (CYS, 98%), 1,8-dimercapto-3,6-dioxactane (DMDO, 98%) and azobisisobutyronitrile (AIBN, 98%) were purchased from Acros (Shanghai, China). Bovine serum albumin (BSA, 96%) was obtained from the American Type Culture Collection (Shanghai, China). AN, AMA and AIBN were purified before use by the traditional method, while other (analytical grade) reagents were used without further purification. The [3-(Methacryloylamino) propyl]dimethyl-(3-sulfopropyl) ammonium hydroxide inner salt (sulfobetaine methacrylamide, SBMAA) was synthesized according to a published procedure [15]. The PAN homopolymer was synthesized by water phase precipitation polymerization in our lab (*M*_η_ = 8.0 × 10^4^ g/mol) [16].

### 2.2. Synthesis and Characterization of P(AN-co-SBMAA-co-AMA)

**Figure 1:**
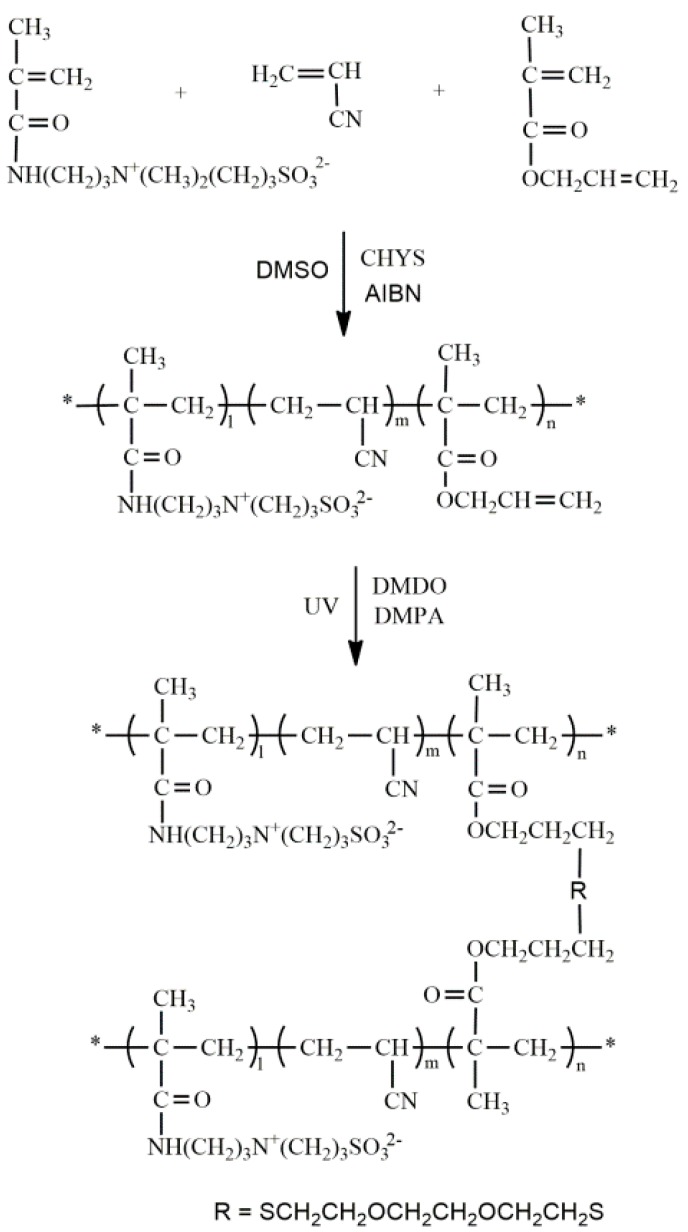
Scheme for the synthesis of P(AN-co-SBMAA-co-AMA). The “*” represent the polymer chain terminal groups.

To prepare crosslinked membranes, the P(AN-co-SBMAA-co-AMA) with pendent unsaturated allyl groups was synthesized firstly via the conventional free-radical copolymerization of the AN, AMA and SBMAA monomers, using AIBN as the initiator, and CYS as the chain transfer agent to avoid gel formation [17]. AN (21.2 g, 0.4 mol), AMA (1 g, 8 mmol), SBMAA (2.23 g, 8 mmol), AIBN (0.35 g, 2.1 mmol), CYS (0.2 g, 1 mmol) and DMSO (250 mL) were introduced into a 500 mL reaction flask. The flask was sealed with a rubber septum, purged with nitrogen gas and placed in an oil bath at 60 °C. The reaction was allowed to run for about 4 h, and stopped before the gel came out. At the end of the reaction, the reaction flask was quenched in cold water, then precipitated into ethanol. The product was then washed three times with ethanol to remove the remaining solvent and monomers. The final product was dried in a vacuum overnight at 50 °C. The synthesis of the P(AN-co-SBMAA-co-AMA) is described in Figure 1.

### 2.3. Preparation of PAN-Based Crosslinked Membranes

PAN-based crosslinked membranes were prepared by the immersion precipitation phase inversion (IPPI) technique. Firstly, P(AN-co-SBMAA-co-AMA) copolymers with pendent allyl groups were dissolved in DMSO at 60 °C to make an 8 wt % homogeneous casting solution. Then, benzoin methyl ether (DMPA) as a photo initiator (2.5 wt %) and DMDO as a crosslinker were added, and the solution was stirred for 15 min at room temperature, then left for 1 h to allow the release of bubbles. The casting solution was spread onto a clean glass plate using a casting knife with a 200 μm gate opening. Next, the nascent membrane was immediately cured with a 300 W high-pressure mercury lamp at a distance of 50 cm for 60 s. Finally, the glass plate was immersed into a coagulation bath of deionized water for 30 min. After peeling off from the glass plate, the membranes were rinsed with ethanol to remove the residual crosslinker, and kept in water until use. To evaluate the impact of crosslinking density on the membranes’ structure and properties, different concentrations of the DMDO (specifically, molar ratios of 0, 0.4, 0.8 and 1.2 with respect to AMA) were applied onto the copolymers. The resulting crosslinked membranes were designated as CM-0, CM-0.4, CM-0.8 and CM-1.2, respectively.

### 2.4. Characterization and Measurement Methods

#### 2.4.1. Characterization of P(AN-co-SBMAA-co-AMA)

The P(AN-co-SBMAA-co-AMA) copolymer composition was checked, by a Nicolet 6700 TA (New Castle, DE, USA) Fourier-transform infrared spectroscopy (FT-IR) spectrometer, with a polymer sample mixed with potassium bromide powder and pressed into a tablet. The copolymer’s chemical structure was further analyzed by an Avance III 500 MHz Bruker (Ettlingen, Germany) ^1^H-NMR spectrometer using DMSO-*d*_6_ as the solvent.

#### 2.4.2. Evaluation of the Crosslinking Density of Membranes

The crosslinking density of the membranes was evaluated by testing their swelling, water pressure resistance and mechanical properties. For the swelling test, each PAN-based crosslinked membrane was cut into five samples of dimension 2 cm × 2 cm (S_0_), and dried in a vacuum oven until the weights stabilized. Then, the membranes were weighed, and the weights were regarded as their initial dry matter (*m*_0_). Subsequently, the membranes were directly immersed into 30 mL of DMSO at 60 °C for 24 h. Next, the membranes were blotted by a filter paper and their dimensions were measured (*S*_f_). Finally, the membranes were gently rinsed with ethanol for three days, and dried in a vacuum oven until the constant weight (*m_f_*) was reached. The gel fraction (*GF*) and dimensional change (Δ*S*) were calculated by the following equations:

*GF* = (*m*_0_ − *m_f_*)/*m*_0_ × 100%

Δ*S* = (*S*_f_ – S_0_)/S_0_ × 100%

The water pressure resistance of the crosslinked PAN membranes was evaluated by a dead-end filtration test. The cut-off membranes were put into a pressure tank without any supporting steel mesh. The pure water flux at different nitrogen pressures was recorded. The sudden increase in the pressure was defined as the rupture pressure (RP). Each RP was obtained by performing three repeated experiments.

The mechanical properties of the crosslinked membranes were measured by a universal testing machine to determine the tensile strength and Young’s modulus in the swollen state at room temperature. Dumbbell-shaped strips (6 mm × 117 mm) were prepared from each sample and soaked in deionized water before testing. Each test was carried out six times. Only the mechanical properties of the swelling membranes were assessed, because the dried membranes were fragile and folded.

#### 2.4.3. Membrane Morphologies

The membrane morphologies were observed by a Carl Zeiss AG supra 55 (Oberkochen, Germany) field-emission scanning electron microscope (SEM) operating at an accelerating voltage of 5 keV. Prior to SEM analysis, the membranes were affixed to a standard sample stub by double-sided carbon conductive tape, and a thin layer of gold was sputtered onto the sample surface.

#### 2.4.4. Permeability Measurements

Water flux was measured using a dead-end filtration module at 0.2 MPa and 25 °C. Before recording the data, the membrane was pressurized for 30 min at 0.25 MPa. The water flux was measured every 15 min continuously for 180 min using the following equation:

*J*_w_ = *V*/A·Δ*t*,

where *J*_w_ is the water flux (L/m^2^h), *V* is the volume of the permeated pure water (L), Δ*t* is the operation time (h) and A is the effective membrane area (1.93 × 10^−4^ m^2^).

The method for the BSA fouling experiment was the same as that for water filtration. Water was replaced by 1 g/L phosphate-buffered saline solution (BSA, pH = 7.4). The protein concentration of the permeation was measured by a UV-Vis spectrophotometer. After protein filtration, the used membranes were immersed in deionized water for 7 h, and the filtration cell was cleaned five times with water. Then, the filtration was started again. The flux rejection and recovery ratio (*FRR*) was calculated according to the following equation, where *J*_b_ is the water flux (L/m^2^h) of the initial membrane for BSA protein filtration and *J*_w_ is the water flux (L/m^2^h) of the washed membrane for pure water:

*FRR*(%) = *J*_b_/*J*_w_ × 100.

## 3. Results and Discussion

### 3.1. Characterization of the P(AN-co-SBMAA-co-AMA) Copolymer

**Figure 2:**
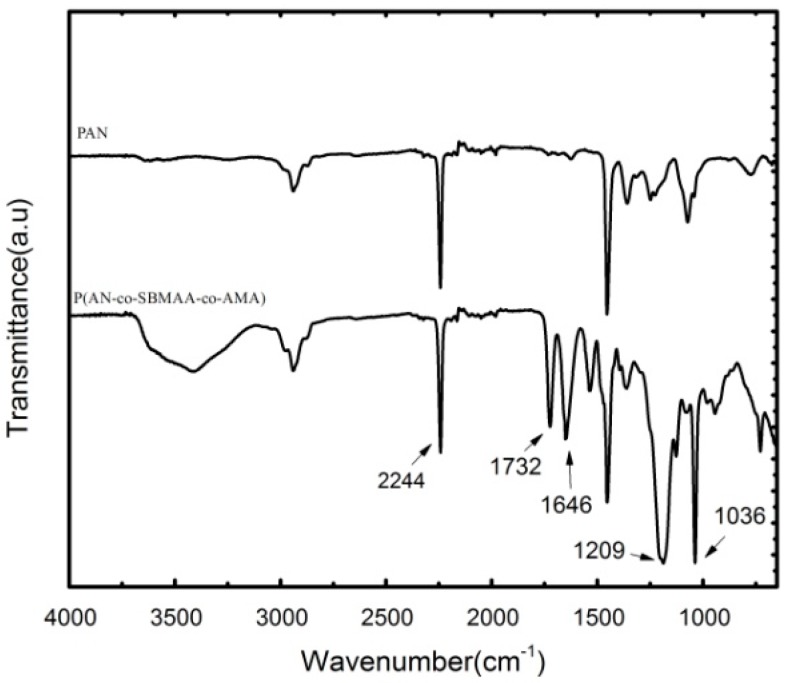
FT-IR spectra of P(AN-co-SBMAA-co-AMA) copolymers.

The structure of the P(AN-co-SBMAA-co-AMA) copolymers with pendant AMA groups is presented by an FT-IR spectrum in Figure 2. The characteristic peak of AN units at 2242 cm^−1^ belongs to –CN groups. It can be seen that, compared with the FT-IR spectrum of pure PAN, P(AN-co-SBMAA-co-AMA) absorption bands at 1732 cm^−1^ should be attributed to the –C=O group in AMA. The characteristic peaks of SBMAA can also be observed at 1646, 1036 and 1209 cm^−1^, which is attributed to the symmetric and asymmetric stretch vibrations of –(SO_3_)^2−^ groups, respectively.

**Figure 3:**
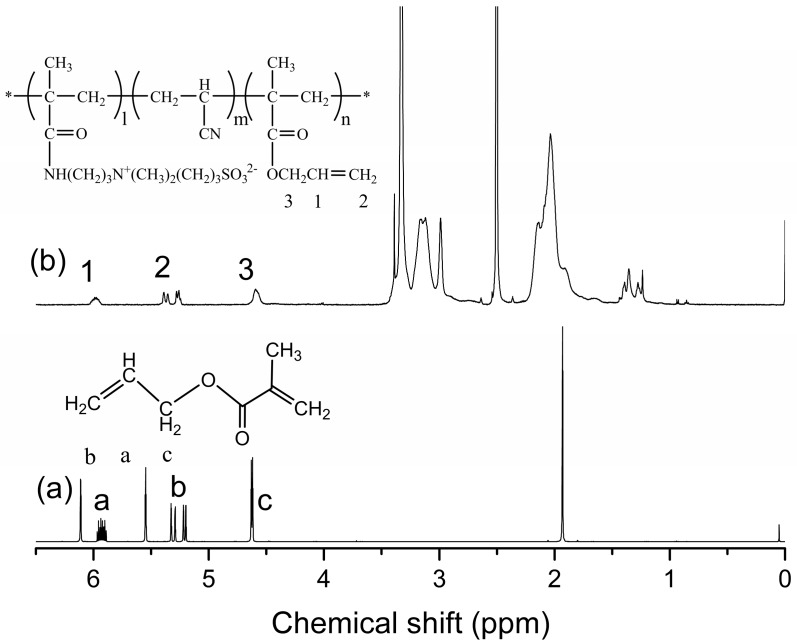
^1^H-NMR spectra of (**a**) monomer AMA, and (**b**) P(AN-co-SBMAA-co-AMA) copolymers.

From the ^1^H-NMR spectra (Figure 3) of AMA, characteristic absorption multiplets at 5.1–5.4 and 5.9 ppm can be attributed to (a) methylene protons and (b) methine protons of the terminal double bond, respectively. Methylene protons (c) of the terminal saturated bond form signals at 4.5 ppm. Signals at 5.5 and 6.1 ppm represent the methylene protons of 2-methylprop-1-ene (–C(CH_3_)=CH_2_). However, the spectra of the P(AN-co-SBMAA-co-AMA) copolymers did not show these signals. It should be noted that AMA has two different types of double bonds: conjugated methacrylic and unconjugated allyl groups. The reactivity of the methacrylic group is quite higher than that of the allyl group because of the presence of the electron-withdrawing ester group [18,19]. Allyl groups inevitably participate in the radical transfer during the polymerization process, but this has little effect on the crosslinking, because the reserved acrylate groups can also participate in the crosslinking reaction. Because of the presence of the –N(CH_3_)^3+^ group in SBMAA, characteristic signals appear at 2.9 ppm. Allyl was introduced into P(AN-co-SBMAA-co-AMA) copolymers, and the reaction occured through the route shown in Figure 1.

### 3.2. Evaluation of Membrane Crosslinking Density

**Figure 4:**
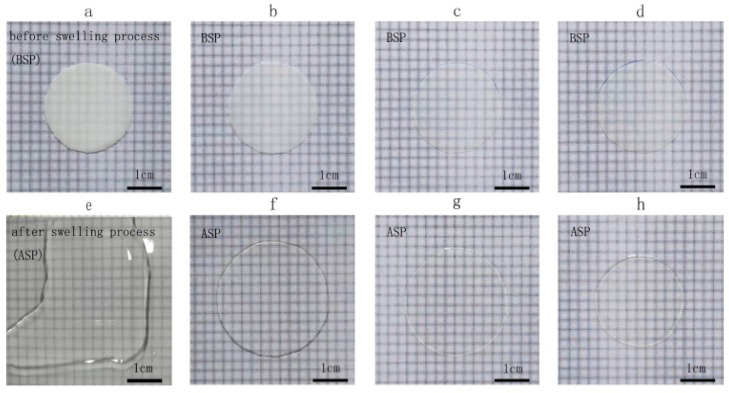
The form of the membranes before (**a**–**d**) and after (**e**–**h**) swelling. (**a**,**e**) CM-0; (**b**,**f**) CM-0.4; (**c**,**g**) CM-0.8; and (**d**,**h**) CM-1.2.

The membrane crosslinking density was evaluated by swelling, tensile strength and water pressure resistance tests. Swelling test results of three crosslinked membranes and the blank PAN membrane are shown in Figure 4. The blank PAN membrane dissolved quickly after being immersed in DMF, while the other crosslinked membranes showed little change. Furthermore, the CM-1.2 membrane kept its shape almost unchanged. Therefore, it can be deduced that the P(AN-co-SBMAA-co-AMA) crosslinking density increased with the use of more DMDO crosslinkers.

Table 1 lists the mechanical and swelling properties of four PAN-based crosslinked membranes. The tensile strength of CM-0 was lower than the three crosslinked membranes, and that of CM-1.2 was the highest. Similarly, the membranes’ RP also improved with an increase in the crosslinking density. Therefore, crosslinking can be used as a traditional technology to improve mechanical properties, because it enhances intermolecular interactions. On the other hand, the blank PAN membrane dissolved easily after being immersed in DMF, while the change in the size (Δ*S*) of the other crosslinked membranes CM-0.4, CM-0.8 and CM-1.2 decreased gradually. Furthermore, CM-1.2 retained almost the same shape as in the initial stage. However, the opposite tendency was observed for GF from Table 1. Therefore, it can be deduced that the P(AN-co-SBMAA-co-AMA) crosslinked owing to the DMDO addition, and the crosslinking density increased by using more DMDO crosslinkers.

### 3.3. Evaluation of Membrane Crosslinking Density

**Figure 5:**
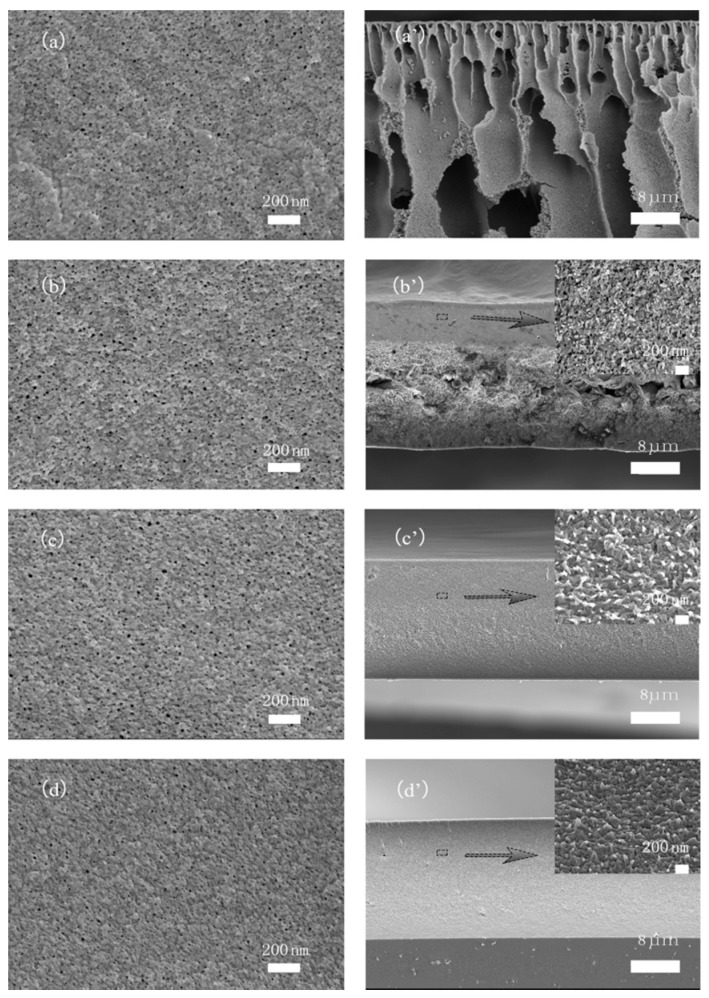
Membrane surface morphology (**a**–**d**) and cross section morphology (**a**′–**d**′). (**a**,**a**′): CM-0; (**b**,**b**′): CM-0.4; (**c**,**c**′): CM-0.8; and (**d**,**d**′): CM-1.2. The arrows represent the partial magnification of the cross section of the membranes.

Figure 5 shows the SEM micrographs of the surfaces and cross-sections of PAN-based membranes. It can be clearly seen that the blank PAN (CM-0) membrane (a), without any crosslinker, exhibits a typical asymmetrical structure consisting of a dense top layer and a finger-like porous sub-layer. While the surface pore size of the crosslinked membranes (b–d) was smaller than that of the blank PAN membrane (a), a conspicuous difference is that the flanked structure of the sub-surface was more compact in the former, and the CM-1.2 membrane—with the most crosslinkers—was denser than the others. Of the three crosslinked membranes, the finger-like pore and sponge-like structure was gradually suppressed and eventually disappeared. The surface pore size of the blank PAN membrane was about 30 nm, while that of the CM-1.2 membrane was about 15 nm.

### 3.4. Permeability Measurements

**Figure 6:**
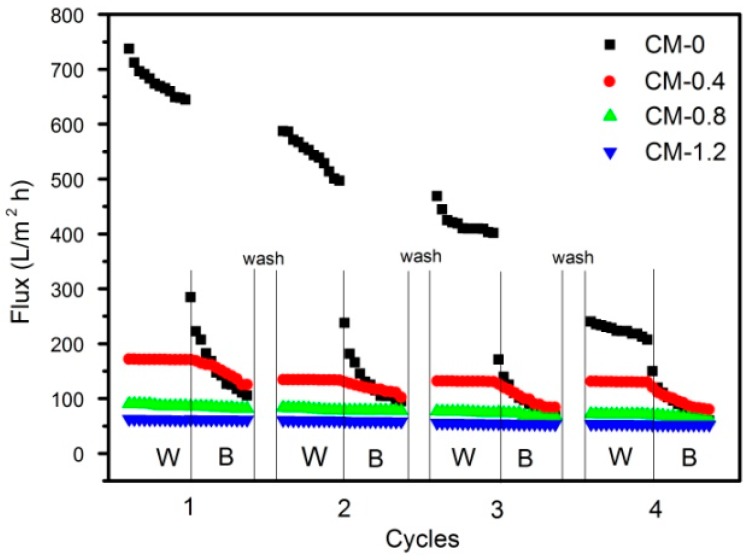
Flux of water and BSA solution for membranes. W: water flux filtration; B: BSA solution filtration.

The pure water flux (*J*_w_), BSA protein flux (*J*_b_) and flux recovery ratio (FRR) of PAN-based membranes are illustrated in Figure 6. The pure water flux decreased dramatically with increasing crosslinking. However, a peculiar phenomenon was observed: if the crosslinked membranes contained zwitterionic groups, then a minor decrease was observed in the BSA protein flux (*J*_b_) compared to that of the pure water flux (*J*_w_). This means that small protein molecules existed in the membranes, which were deposited and adsorbed on the membrane surface. The CM-0.4, 0.8 and 1.2 membranes contained enough zwitterionic groups to result in a high flux-recovery ratio (FRR) even after three cycles of BSA solution ultrafiltration. An obvious reason is that the hydrophilic zwitterionic group, SBMA, with a balanced charge and minimized dipole, is an excellent candidate for nonfouling materials because of its strong hydration capacity owing to electrostatic interactions [20,21].

## 4. Conclusions

In conclusion, crosslinked PAN membranes with different crosslinking densities were prepared by a facile thiol-ene click reaction under UV irradiation. By regulating the crosslinking densities, the membranes were presented with different topological structures. It was found that the mechanical properties of the membranes greatly improved after crosslinking. Moreover, the crosslinked membranes can maintain their shape well, even when soaped into DMF solution. It was also found that the introduction of betaine, a zwitterionic sulfonic acid, significantly improved the antifouling properties of the separation membranes. Therefore, crosslinked membranes can be used for high-pressure operation and for treating wastewater containing high concentrations of organic waste.

## Figures and Tables

**Table 1:** Mechanical and swelling properties of the membranes.

Membrane	Tensile Strength (MPa)	Young’s Modulus (MPa)	*RP* (MPa)	Δ*S* (%)	*GF* (%)
CM-0	0.36	18.9	0.01	/	0
CM-0.4	0.96	19.1	0.05	34.3	81.8
CM-0.8	1.25	24.4	0.08	25.2	91.3
CM-1.2	1.64	33.4	0.11	1.5	94.6
